# Depression, Anxiety and Antidepressants and Anxiolytics Use in Spanish Informal Caregivers according to the Physical Activity Frequency: EHSS 2014–2020

**DOI:** 10.3390/healthcare11070990

**Published:** 2023-03-30

**Authors:** Angel Denche-Zamorano, Yeray Rodriguez-Redondo, Sabina Barrios-Fernandez, María Mendoza-Muñoz, Jorge Rojo-Ramos, Miguel Angel Garcia-Gordillo, Jose C. Adsuar, Laura Muñoz-Bermejo

**Affiliations:** 1 Promoting a Healthy Society Research Group (PHeSO), Faculty of Sport Sciences, University of Extremadura, 10003 Caceres, Spain; 2 Social Impact and Innovation in Health (InHEALTH), University of Extremadura, 10003 Caceres, Spain; 3 Occupation, Participation, Sustainability and Quality of Life (Ability Research Group), Nursing and Occupational Therapy College, University of Extremadura, 10003 Cáceres, Spain; 4 Research Group on Physical and Health Literacy and Health-Related Quality of Life (PHYQOL), Faculty of Sport Sciences, University of Extremadura, 10003 Cáceres, Spain; 5 Departamento de Desporto e Saúde, Escola de Saúde e Desenvolvimento Humano, Universidade de Évora, 7004-516 Évora, Portugal; 6 Physical Activity for Education, Performance and Health, Faculty of Sport Sciences, University of Extremadura, 10003 Caceres, Spain; 7 Universidad Autónoma de Chile, Talca 3467987, Chile

**Keywords:** nursing, healthcare, psychology, physical exercise, medication

## Abstract

Depression and anxiety are two of the most common mental diseases both in formal and nonformal caregivers. Physical activity during leisure time seems to have benefits on their practitioner’s mental health. This study aimed to analyze the associations between physical activity frequency (PAF) and depression and anxiety status, as well as antidepressant and anxiolytic use in Spanish nonformal caregivers. A cross-sectional study with data from the 2014 and 2020 European Health Interview Surveys in Spain (EHSS) including 4520 Spanish nonformal caregivers was carried out. The PAF was found to be related to depression and anxiety, as well as antidepressants and anxiolytics use (*p* < 0.001), with the highest proportions of these variables found in the inactive population (*p* < 0.05), while the active and very active populations showed the lowest proportions (*p* < 0.05). Weak but statistically significant correlations were found between all variables of interest (*p* < 0.001). Being female, older, and dedicating more hours per week to caregiving and caring for nonfamily members were found to have increased risks of depression, anxiety and antidepressants or anxiolytics use. Nonformal caregivers who were not physically active during their leisure time had higher mental disorders and psychotropic drug use proportions than the active and very active caregivers. Thus, increasing nonformal caregivers’ PAF could be a protective tool.

## 1. Introduction

In Europe, mental health is a major public health concern in terms of prevalence, burden of disease and disability [1]. Mental health disorders include a wide range of conditions that negatively alter mood, cognition and behavior [2], affecting more than one-third of the European population and showing an upward trend in the degree of involvement [3]. Mental health problems, moreover, rank second in terms of disease burden, with 19% of the total. In addition, in terms of disability in many countries, depression, anxiety and schizophrenia are the leading cause [3].

Depression is a common mental disorder affecting approximately 3.8% of the population, making it the world’s leading cause of disability and a major contributor to the overall global burden of disease [3]. Its symptoms are associated with a depressed mood or a loss of enjoyment or interest in activities; for it to be considered depression, it must be prolonged in time for at least two weeks, most of the day, almost every day. In addition, it is associated with other psychological symptoms [4], and it is a serious problem especially when it is recurrent and of high/moderate intensity and can even lead to suicide [5]. It has been observed that immigrants living in Spain have a higher prevalence of suffering from depression or anxiety compared to the native population [6]. We also found that women have a higher rate of depression and anxiety than men in Spain [7]. This pathology is usually combated with psychological treatments and/or antidepressant drugs, such as selective serotonin reuptake inhibitors (SSRIs) and tricyclic antidepressants [4]. Anxiety disorders are also very common in today’s population, with an estimated 301 million people suffering from them in 2019, including 58 million adolescents and children [8]. This pathology is characterized by excessive worry and fear, in addition to associated behavioral disorders [9]. Thus, there are psychological treatments for this pathology, which in most cases are effective, but depending on the age and severity, anxiolytic intake can be also considered [9].

One of the roles with a high risk of suffering psychological problems is caregiver, regardless of the pathology of the dependent person. There are formal and informal caregivers, the former a person not necessarily from the family of the care recipient, who may or may not be trained to perform his or her role but receives financial compensation, and the latter, a person from the social network of the care recipient, who perform this role on an unpaid and voluntary manner [10]. In European countries, we find that between 11% and 17% of the population is in the role of informal caregiver, and this figure rises to approximately 20% in the US population. Caregivers develop important physical, psychological and emotional burdens, affecting their health [11]. Between 27 and 40% of caregivers in Spain have impaired mental health (anxiety and depression), and 33.1% of nonprofessional caregivers have high emotional distress [12,13]. Approximately 41.5% of caregivers of elderly people with dementia have made a medical or psychiatric consultation because of psychological problems caused by their caregiving role, which are usually frustration and helplessness, anxiety, irritability, anger, depression and sadness, among other symptoms [11,14]. Moreover, their physical health is also affected, with 57.5% of caregivers reporting problems such as fatigue, stress, back and joint pain and insomnia, with 65.6% of them attending a medical consultation [11].

The amount of PA beneficially affects quality of life, with a connection between PA, mental health and physical condition. Therefore, PA is associated with improved mental health [15,16] and positive effects on depression, while longer sedentary time is related to poorer mental health [17]. These positive effects on mental health are based on the fact that the body responds to PA by secreting endorphins to the brain, which reduce pain and cause general euphoria [18]. The association between the release of endorphins and a reduction in the symptoms of anxiety and depression has been proven [18,19,20]. Then, although PA is considered an effective approach to combat symptoms of these mental disorders [20,21,22], it is needed to prove a causal relationship between PA and mental health [21].

We also found previous studies exploring the ability of PA to improve psychological aspects such as successful coping, self-esteem and reduced stress in a population of informal caregivers [22]. On the other hand, we found an improvement in the quality of life of informal caregivers who have greater physical strength [23]; this improved physical condition allows them to carry out their caregiving tasks more efficiently, positively affecting their psychological state. However, there are numerous barriers that may influence the practice of physical activity in informal caregivers [24]. These may include lack of time, emotional burden, physical fatigue, lack of resources, lack of support, health problems or lack of knowledge [24].

Therefore, the present study aimed to explore, in a sample of Spanish informal caregivers, (1) the prevalence of depression and anxiety and the consumption of antidepressants and anxiolytics concerning sex; (2) the possible differences in the proportions of the prevalence of depression and anxiety according to the frequency of PA; and (3) the probability of presenting depression or anxiety according to the frequency of PA. These objectives were based on the hypotheses that (1) the prevalence of depression and anxiety will be sex-dependent in the population of Spanish informal caregivers, and we predict that female caregivers will present more mental health disturbances than male caregivers; (2) differences will be found in the prevalence proportions of anxiety and depression according to the PAF, and people who are frequently physically active will present less mental health disturbances; and (3) there will be a greater probability of presenting a risk of depression or anxiety in Spanish informal caregivers with a lower PAF.

## 2. Materials and Methods

### 2.1. Design and Data Source

The public files of the European Health Surveys in Spain 2014 (EHSS 2014) and 2020 (EHSS 2020) were the data sources for this cross-sectional study of adult nonformal caregivers residing in Spain. The EHSS is included in the European Statistical System, being a survey of compulsory application in all Member States for the measurement in a harmonized way of the health status, lifestyles and other health determinants and use of health services of EU citizens. The EHSS 2014 and EHSS 2020 were regulated by Commission Regulation (EU) 141/2013 of 19 February 2013 and Commission Regulation (EU) 255/2018 of 19 February 2019, implementing Framework Regulation 1338/2008 [25,26]. Supervised by the statistical office of the European Union, Eurostat, the EHSS 2014 and EHSS 2020 were conducted by the National Institute of Statistics in collaboration with the Spanish Ministry of Health. The data were published by the INE in anonymous files for public access and use, being considered nonconfidential data and not requiring approval by any accredited ethics committee.

### 2.2. Variables

The data for the variables of interest were extracted from the public files of the EHSS 2014 and EHSS 2020. The data were extracted for the following variables:

Sex (data from item “SEXOa”: Man or Woman);

Age (data from item “EDADa”: Years);

Depression status (data from item Q.25a.20: “Have you ever suffered from depression?”, with the possible answers of “Yes”, “No”, “Don’t know” or “No answer”);

Anxiety status (data from item Q.25a.21: “Have you ever suffered from chronic anxiety?”, with the possible answers of “Don’t know” or “No answer”);

Anxiolytics use (data from items: Q.85 (“During the last 2 weeks, have you taken any medications that were prescribed to you by a doctor?”, “Yes” or “No”) and Q.87a.7 (“Next, I am going to read you a list of medications, please tell me which one(s) of them you have taken in the last 2 weeks: tranquillizers, relaxants, sleeping pills?”, “Don’t know” or “No answer”); it was considered as “Yes” when the participant answered “Yes” to item Q.87a.7, “No” when the participant answered “Yes” to item Q.87a.7, “No” when the participant answered “No” to item Q.87a.7, and “No” when the participant answered “No” to item Q.85 or with “Yes” to item Q.85 and “No” to item Q.87a.7;

Antidepressants use (data from items Q.85 and Q.87a.7 (“Next I am going to read you a list of types of medications, please tell me which one(s) of them you have consumed in the last 2 weeks: antidepressants, stimulants?”, “Don’t know” or “No answer”); it was considered as “Yes” when the participant answered “Yes” to item Q.87a.14 and “No” when the participant answered “No” to item Q.85 or with “Yes” to item Q.85 and “No” to item Q.87a.14;

Physical activity frequency (PAF) (data from item Q.112: “Which of these possibilities best describes the frequency with which you do some physical activity in your free time?” “I spend my free time almost completely sedentary (reading, watching TV, going to the movies, etc.)”. “I do some occasional PA or sports (walking or cycling, gardening, gentle gymnastics, recreational activities that require light exertion, etc.)”. “I do PA several times a month (sports, gymnastics, running, swimming, cycling, team games, etc.)”. “I do sports or physical training several times a week”. For this study, these responses were labeled as: “Never”, “Occasional”, “Active” and “Very active”);

Nonformal caregiver status (data from item Q.133: “Do you care, at least once a week, for an elderly person or someone who has a chronic disease?” Do not consider it if it is part of your job; having as possible answers: “Yes”, “No”, “Don’t know” or “No answer”);

Who do you care for (data from item Q.134: “The person(s) you care for are relatives”, “other persons”, “Don’t know” or “No answer”);

Hours of care per week (data from item Q.134: “In total, how many hours per week do you spend caring for this/these person(s)?” “Less than 10 h per week”, “10 h or more per week but less than 20”, “20 h per week or more”, “Don’t know” or “No answer”).

### 2.3. Participants

Following Regulations (EU) No. 141/2013 and No. 255/2018; the EHSS 2014 and EHSS 2020 had as a target population persons aged 15 and over residing in Spain. Both used a stratified three-stage random sampling system, in which, firstly, the census units were randomly selected, within them, family dwellings, and from these, one of their residents aged 15 and over. This sampling system, as well as the sample calculation, data processing, the treatment of errors or missing data and the interview procedure, is fully explained in the methodologies of the EHSS 2014 and EHSS 2020.

**Figure 1:**
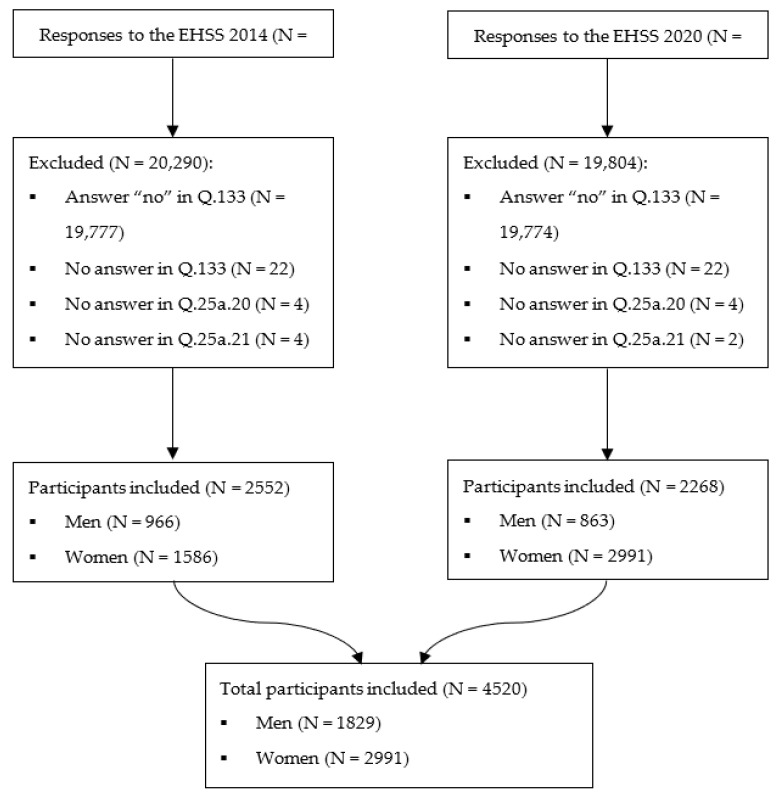
Selection process flowchart.

These EHSS presented a final sample of 44,914 participants as a whole, 22,842 (EHSS 2014) and 22,072 (EHSS 2020), who were interviewed by previously trained and accredited interviewers between January 2014 and January 2015 (EHSS 2014) and between July 2019 and July 2020 (EHSS 2020). For this research, the following were established as inclusion criteria: being a nonformal caregiver (having answered “Yes” for item Q.133), submitting data to the questions on depression (Q.25a.20), chronic anxiety (Q.25a.21), anxiolytic medication (Q.87a.7), antidepressant medication use (Q.87a.14) and PA (Q.112). Thus, 39,551 noncaregiver participants were excluded due to the fact of answering “No” for item Q.133 (EHSS 2014: 19,777; EHSS 2020: 19,774); 44 participants with no data for that same item (EHSS 2014: 22; EHSS 2020: 22); 8 participants with no data on depression for Q.25a.20 (EHSS 2014: 4; EHSS 2020: 4); 6 participants with no data on anxiety for Q.25a.21 (EHSS 2014: 4; EHSS 2020: 2); 1 participant with no data on anxiolytic use (EHSS 2014); and 2 participants with no data on antidepressant use (EHSS 2014: 1; EHSS 2020: 1). Therefore, the final sample included 4520 nonformal adult caregiver participants residing in Spain (EHSS 2014: 2552; EHSS 2020: 2268): 1829 men (EHSS 2014: 966; EHSS: 863) and 2991 women (EHSS 2014: 1586; EHSS 2020: 2991). The characterization of the sample according to age, PAF, caregiving hours and people they cared for, and by sex can be found in Table S1. Figure 1 shows the exclusion process followed to select the final sample.

### 2.4. Statistical Analysis

First, the data normality was studied with the Kolmogorov–Smirnov test. A descriptive analysis was performed presenting the sample according to the variables depression status, anxiety status, and antidepressants and anxiolytics use in absolute and relative frequencies, both for the total sample and by sex. A pairwise z-test was performed for independent proportions to test for possible differences in the proportions between sexes. In addition, the dependence relationships between the aforementioned variables and sex were studied using the chi-square test, evaluating the strength of these relationships with the contingency coefficient. These same tests were used to analyze the dependence relationships between the above variables and the PAF, as well as the possible differences in proportions between the different PAF groups. The odds ratios (ORs) of presenting depression, anxiety, or antidepressants or anxiolytics use were calculated as a function of the PAF, taking the inactive group as a reference. A correlation study was carried out calculating Spearman’s rho between the variables PAF, depression and anxiety status, antidepressants and anxiolytics use, sex and age, applying Bonferroni correction. Finally, multiple binary logistic regression models were performed taking as the dependent variables depression and anxiety status and antidepressants and anxiolytic use, and as independent variables PAF, sex, age, hours of care and caregiver.

IBM SPSS Statistical v.25 software was used to perform all statistical analyses. The statistical significance level was <0.05.

## 3. Results

Insufficient evidence was found to assume that the data followed a normal distribution, *p* < 0.001. Table 1 shows the depression (15.0% and 11.8%) and anxiety proportions (13.8% and 11.4%), and antidepressant (7.6% and 6.7%) and anxiolytic use (16.4% and 13.5%) in Spanish nonformal caregivers from the EHSS 2014 and EHSS 2020. In all of the above variables, women presented higher proportions than men (*p* < 0.05) in both surveys (Table 1). Similarly, dependency relationships were found in both surveys between sex and depression and anxiety status and antidepressants and anxiolytic use (*p* < 0.001) (Table 1).

**Figure 2:**
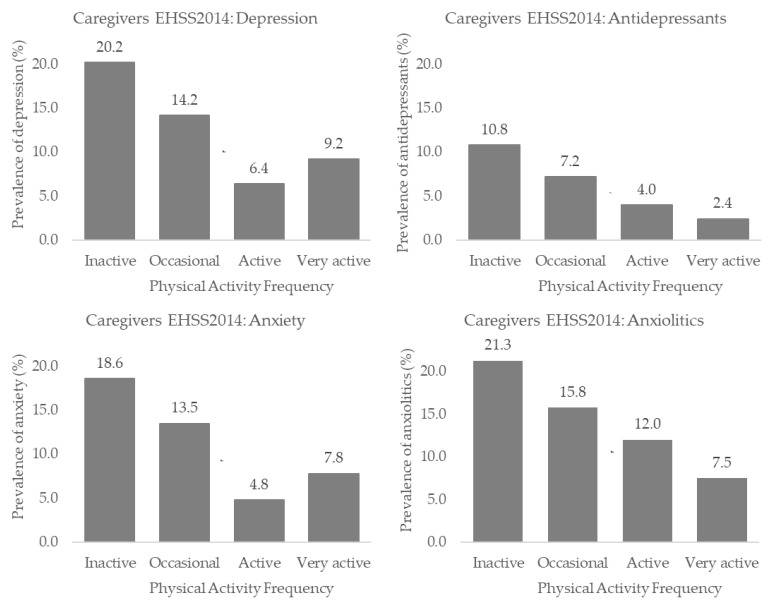
Depression and anxiety and antidepressants and anxiolytics use proportions according to the physical activity frequency in Spanish informal caregivers from the EHSS 2014.

**Figure 3:**
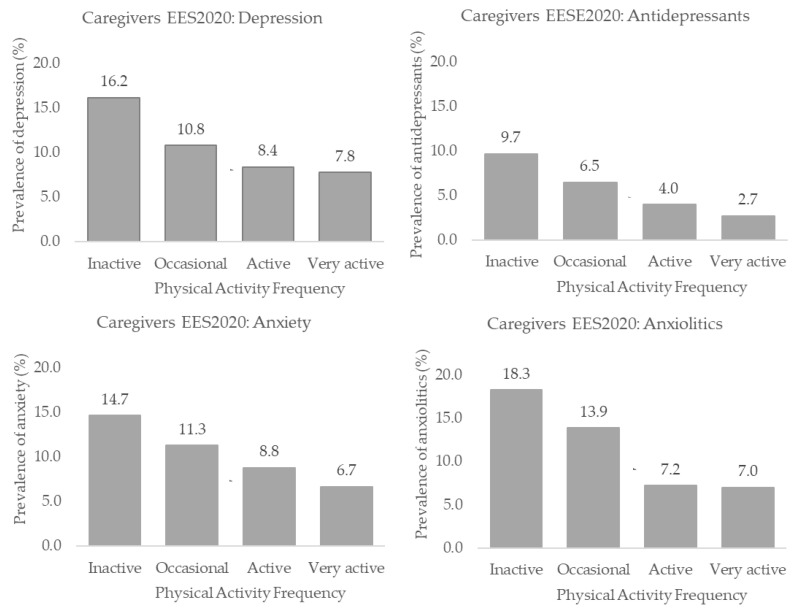
Depression, anxiety and antidepressants and anxiolytics use proportions according to the physical activity frequency in Spanish informal caregivers from the EHSS 2020.

Table S2 and Figure 2 and Figure 3 show the depression, anxiety and antidepressant and anxiolytic use proportions according to the PAF in the EHSS 2014 and the EHSS 2020. In both surveys, the highest depression, anxiety and antidepressant and anxiolytic use proportions were higher in the inactive population than in the active and very active individuals (*p* < 0.05) (Table S2). In both the EHSS 2014 and the EHSS 2020, dependency relationships were found between PAF and the rest of the variables (*p* < 0.001) (Table S2).

In both the EHSS 2014 and the EHSS 2020, lower odds ratios for depression, anxiety and antidepressants and anxiolytics use were found in the very active population compared to the inactive population (Table 2). These odds ratios are also shown in Figures S1 and S2.

Weak, although statistically significant correlations, were found between PAF and depression, anxiety and antidepressants and anxiolytics use proportions (*p* < 0.001), both in the EHSS 2014 and the EHSS 2020 (Table 3).

Table S3 shows the multiple binary logistic regression models related to the depression status (explaining 7.4% of the variance, Nagelkerke’s R^2^), anxiety status (Nagelkerke’s R^2^ = 7.7%), antidepressants use (Nagelkerke’s R^2^ = 7.3%) and anxiolytics’ use (Nagelkerke’s R^2^ = 9.0%) according to the EHSS 2014 data. Being female, presenting older age, being physically inactive, dedicating a greater number of hours per week to caregiving, and caring for nonfamily members were presented as increased risks for depression (Table S3). These same variables, except the caregiver one, were presented as risks for anxiety, antidepressant use, and anxiolytic use (Table S3). Table S4 presents these same models with data from the EHSS 2020, with a Nagelkerke’s R^2^ of 5.4% (depression status), 3.9% (anxiety status), 5.5% (antidepressants use) and 6.6% (anxiolytics use). According to data from this survey, being female, older, inactive, and dedicating more hours per week to caregiving and caring for a nonfamily member were presented as risks for depression (Table S4). In contrast, being female and physically inactive were found to be risks for anxiety (Table S4). For antidepressants and anxiolytic use, being female, older and inactive were presented as risks (Table S4).

## 4. Discussion

The main findings of the study show that both in 2014 and 2020 female caregivers suffered more anxiety and depression and consumed more drugs related to these pathologies (anxiolytics and antidepressants) than male caregivers. Different studies confirm the trend of these results and the association between sex and anxiety and depressive symptoms [27,28]. Female caregivers presented a higher risk of moderate to severe depressive and anxiety symptoms and reported significantly higher levels of perceived stress, depression and anxiety than male caregivers [29]. This association can be explained by the stress and physical burnout of caregiving aspects, as female caregivers present a greater caregiving burden, and this contributes to the occurrence of mood disorders [30]. Furthermore, concerning the use of anxiolytics and antidepressants, and in line with the results obtained, women were also more likely to use medication than men [31]. It is possible that these figures may vary, as it was found that more than half of the people with mental morbidity had never been diagnosed with a mental disorder. We understand that the result obtained regarding mental health status and the sex of the caregiver could be due to the perceived social support in the different sexes, as has also been mentioned in other studies.

Another finding of this study was that both depression and anxiety were more frequent in inactive caregivers. In the same sense, anxiolytics and antidepressants use were higher in nonphysically active caregivers. Thus, caregivers who did not engage in PA suffered from mental health disorders and consumed drugs decreased in 2020 compared to 2014. In this line, several studies have shown an association between PA and mental health status related to symptoms of depression [32] and anxiety [33] in caregivers [34,35,36]. A higher PA is associated with better mental health status, and walking improves symptoms compared to inactivity, although activity at a moderate and/or vigorous intensity is an even greater improvement [22]. In addition, caregivers with more periods of inactivity experience higher levels of stress, while those with shorter periods of inactivity have a better quality of life, in general, and report better physical and psychological well-being [37]. A good level of mental health in caregivers could be achieved with a balance between caregiving and self-care activities, where physical activity could contribute to improving stress.

The risk of suffering anxiety and depression increases in caregivers who perform occasional PA concerning those who perform it more frequently. These data evolved from 2014 to 2020, such that in 2014 occasional PA performed by caregivers only influenced depressive symptoms, but in 2020 PA improved depression, anxiety and drug use. In this sense, helping caregivers to be physically active may improve their health status and self-perception, feel better not only about themselves but also about their day-to-day caregiving situation, and may help caregivers maintain their caregiving roles for longer periods with less risk of suffering alterations related to their mental health and quality of life [38,39,40].

Associations were found between PAF and age, anxiety and depression and anxiolytics and antidepressants use in 2014 and 2020, although in 2014 the association was weak between PAF and sex (women), and in 2020 this association was not found. Performing PA could be a factor in alleviating symptoms related to anxiety and/or depression in caregivers, and it could even decrease drug use related to these pathologies. These findings suggest that healthcare workers should focus on caregivers, especially women, and perform individualized interventions aimed at the early detection and prevention of mental diseases and routinely offer PA interventions to informal caregivers.

This study has certain limitations: (1) We were unable to differentiate between being a caregiver of an adult and of a child and, therefore, the association between anxiety, depression and drug use and PA among adult or child caregivers. (2) Neither mental disorders nor drugs consumed were analyzed concretely due to the lack of access to participants’ medical records. (3) The overload data and differences between PAF and caregiver burden were not analyzed, so relationships between both variables and mental health could not be established, which should be considered in future research.

## 5. Conclusions

Mental health disorders, such as anxiety and depression, are more frequent in female informal caregivers. Consequently, anxiolytics and antidepressant consumption are also more frequent in women.

An increase in the PAF of informal caregivers could improve anxiety and depressive symptoms and drug use. In addition, inactive caregivers showed a higher risk of emotional disturbances, such as anxiety and depression. Therefore, PA interventions in informal caregivers could prevent and/or improve mental health.

## Figures and Tables

**Table 1:** Depression, anxiety, antidepressant use and anxiolytic use proportions in Spanish informal caregivers, as well as sex comparison (EHSS2014–2020).

EHSS 2014										
	Total		Men		Women					
	N = 2552	(%)	*n* = 966	(%)	*n* = 1586	(%)	x^2^	df	*p*	CC
Depression	383	(15.0)	88	(9.1)	295	(18.6) *	42.4	1	<0.001	0.128
No depression	2169	(85.0)	878	(90.9)	1291	(81.4) *				
Anxiety	352	(13.8)	75	(7.8)	277	(17.5) *	47.5	1	<0.001	0.135
No anxiety	2200	(86.2)	891	(92.2)	1309	(82.5) *				
Antidepressant	195	(7.6)	39	(4.0)	156	(9.8) *	28.6	1	<0.001	0.105
No antidepressant	2357	(92.4)	927	(96.0)	1430	(90.2) *				
Anxiolytics	419	(16.4)	94	(9.7)	325	(20.5) *	50.7	1	<0.001	0.140
No anxiolytics	2133	(83.6)	872	(90.3)	1261	(79.5) *				
**EHSS 2020**										
	**Total**		**Men**		**Women**					
	***n* = 2268**	**(%)**	***n* = 863**	**(%)**	***n* = 1405**	**(%)**	**x^2^**	**df**	***p***	**CC**
Depression	268	(11.8)	72	(8.3)	196	(14.0) *	16.1	1	<0.001	0.084
No depression	2000	(88.2)	791	(91.7)	1209	(86.0) *				
Anxiety	258	(11.4)	60	(7.0)	198	(14.1) *	27.0	1	<0.001	0.109
No anxiety	2010	(88.6)	803	(93.0)	1207	(85.9) *				
Antidepressant	151	(6.7)	32	(3.7)	119	(8.5) *	19.5	1	<0.001	0.092
No antidepressants	2117	(93.3)	831	(96.3)	1286	(91.5) *				
Anxiolytics	306	(13.5)	82	(9.5)	224	(15.9) *	19.0	1	<0.001	0.091
No anxiolytics	1962	(86.5)	781	(90.5)	1181	(84.1) *				

*n* (participants); % (percentage); x^2^ (Pearson chi-square); df (degree freedom); *p* (*p*-value from chi-square test); CC (contingency coefficient); * (significant differences in a pairwise z-test for independent proportions, *p* < 0.05).

**Table 2:** Odds ratios of depression, anxiety and antidepressants and anxiolytics use according to the physical activity frequency in Spanish informal caregivers (EHSS2014–2020).

EHSS 2014										
	Inactive	Occasional			Active			Very Active		
		OR	95% CI		OR	95% CI		OR	95% CI	
Depression	Ref.	0.65 *	0.52	0.83	0.27 *	0.16	0.46	0.40 *	0.26	0.61
Anxiety	Ref.	0.68	0.32	1.47	0.22 *	0.08	0.63	0.37 *	0.15	0.90
Antidepressants	Ref.	0.64	0.24	1.72	0.34	0.11	1.12	0.20 *	0.05	0.85
Anxiolytics	Ref.	0.69	0.34	1.42	0.50	0.23	1.09	0.30 *	0.12	0.73
**EHSS 2020**										
	**Inactive**	**Occasional**			**Active**			**Very Active**		
		**OR**	**95% CI**		**OR**	**95% CI**		**OR**	**95% CI**	
Depression	Ref.	0.63 *	0.47	0.84	0.48 *	0.29	0.77	0.44 *	0.29	0.67
Anxiety	Ref.	0.74 *	0.55	0.99	0.56 *	0.35	0.91	0.42 *	0.27	0.66
Antidepressants	Ref.	0.65 *	0.45	0.93	0.39 *	0.20	0.76	0.26 *	0.13	0.51
Anxiolytics	Ref.	0.72 *	0.55	0.94	0.35 *	0.21	0.58	0.34 *	0.22	0.52

OR (odds ratios (ORs) > 1 indicate a higher risk of reporting depression, anxiety and antidepressant or anxiolytic use); 95% CI (95% confidence interval of the odds ratio); * (*p*-value < 0.05); Ref. (reference).

**Table 3:** Spearman’s bivariate correlation between the depression and anxiety proportions and physical activity frequency, age and sex (EHSS2014-EHSS2020).

EHSS2014							
	PAF	Depression	Anxiety	Antidepressants	Anxiolytics	Age	Sex
	rho	rho	rho	rho	rho	rho	rho
PAF	1.000	−0.125 **	−0.124 **	−0.107 **	−0.119 **	−0.153 **	−0.087
Depression	−0.125 **	1.000	0.554 **	0.523 **	0.385 **	0.100 **	−0.129 **
Anxiety	−0.124 **	0.554 **	1.000	0.480 **	0.433 **	0.075 **	−0.136 **
Antidepressants	−0.107 **	0.523 **	0.480 **	1.000	0.474 **	0.068	−0.106 **
Anxiolytics	−0.119 **	0.385 **	0.433 **	0.474 **	1.000	0.152 **	−0.141 **
Age	−0.153 **	0.100 **	0.075 **	0.068	0.152 **	1.000	0.026
Sex	−0.087 **	−0.129 **	−0.136 **	−0.106 **	−0.141 **	0.026	1.000
**EHSS2020**							
	**PAF**	**Depression**	**Anxiety**	**Antidepressants**	**Anxiolytics**	**Age**	**Sex**
PAF	1.000	−0.099 **	−0.088 **	−0.101 **	−0.125 **	−0.110 **	−0.057
Depression	−0.099 **	1.000	0.463 **	0.549 **	0.367 **	0.084 **	−0.084 **
Anxiety	−0.088 **	0.463 **	1.000	0.411 **	0.375 **	0.014	−0.109 **
Antidepressants	−0.101 **	0.549 **	0.411 **	1.000	0.417 **	0.054	−0.093 **
Anxiolytics	−0.125 **	0.367 **	0.375 **	0.417 **	1.000	0.119 **	−0.092 **
Age	−0.11 0 **	0.084 **	0.014	0.054	0.119 **	1.000	0.026
Sex	−0.057	−0.084 **	−0.109 **	−0.093 **	−0.092 **	0.026	1.000

PAF: physical activity frequency; rho (Spearman’s correlation coefficient with Bonferroni correction factor); ** *p* < 0.001; Sex (Reference: Women).

## Data Availability

Datasets are available upon reasonable request to the corresponding author. Microdata were obtained from the website of the National Statistics Institute: https://www.ine.es/ftp/microdatos/enceursalud/datos_2014_hogares.zip (accessed on 7 January 2023); https://www.ine.es/ftp/microdatos/enceursalud/datos_2020_hogar.zip (accessed on 7 January 2023).

## Supplementary Materials

The following supporting information can be downloaded at: https://www.mdpi.com/article/10.3390/healthcare11070990/s1, Figure S1: Risk probability of depression, anxiety, antidepressant use and anxiolytic use in the inactive population according to the physical activity frequency of Spanish nonformal caregivers from EHSS 2014 (ORs: odds ratios; CI: confidence interval); Figure S2: Odds ratios of depression, anxiety and antidepressants and anxiolytics use in the inactive population based on physical activity frequency in Spanish nonformal caregivers from EHSS 2014 (ORs: odds ratios; CI: confidence interval); Table S1: Sample characterization; Table S2: Depression, anxiety and antidepressants and anxiolytic use proportion in Spanish nonformal caregivers according to the physical activity frequency from EHSS2014–2020; Table S3: Logarithmic binary regression model for depression, anxiety and antidepressants and anxiolytics use from EHSS2014; Table S4: Logarithmic binary regression model for depression, anxiety and antidepressants and anxiolytics use (EHSS2020).

## References

[1] World Health Organization—Regional Office for Europe (2015). The European Mental Health Action Plan 2013–2020.

[2] Litin S.C., Litin S.C. (2018). Mayo Clinic Family Health Book.

[3] Ministerio de Sanidad; Servicios Sociales e Igualdad; Instituto Nacional de Estadística Encuesta Nacional de Salud España 2017. Informe Monográfico de Salud Mental. SG Información Sanitaria Ministerio de Sanidad, Consumo y Bienestar Social 2017, 21–25. https://www.sanidad.gob.es/estadEstudios/estadisticas/encuestaNacional/encuestaNac2017/SALUD_MENTAL.pdf.

[4] Organización Mundial de la Salud Depresión. https://www.who.int/es/news-room/fact-sheets/detail/depression.

[5] Melhem N.M., Porta G., Oquendo M.A., Zelazny J., Keilp J.G., Iyengar S., Burke A., Birmaher B., Stanley B., Mann J.J. (2019). Severity and Variability of Depression Symptoms Predicting Suicide Attempt in High-Risk Individuals. JAMA Psychiatry.

[6] Romero-Acosta K., Penelo E., Noorian Z., Ferreira E., Domènech-Llaberia E. (2014). Racial/Ethnic Differences in the Prevalence of Internalizing Symptoms: Do Latin-American Immigrant Show More Symptomatology than Spanish Native-Born Adolescents?. J. Health Psychol..

[7] Sanchez-Garcia M., De La Rosa-Caceres A., Stasik-O’Brien S., Mancheno-Barba J.J., Lozano O.M., Diaz-Batanero C. (2021). Norms According to Age and Gender for the Spanish Version of the Inventory of Depression and Anxiety Symptoms (IDAS-II). Front. Psychol..

[8] Institute for Health Metrics and Evaluation Global Health Data Exchange (GHDx). https://ghdx.healthdata.org/.

[9] Organización Mundial de la Salud Trastornos Mentales. https://www.who.int/es/news-room/fact-sheets/detail/mental-disorders.

[10] Córdoba A.M.C., Aparicio M.J.G. (2014). Efectos de cuidar personas con Alzheimer: Un estudio sobre cuidadores formales e informales. Pensam. Psicológico.

[11] Artaza Artabe I., Primitivo Ramos C., González Nuñez J., Martínez Hernández D. Estudio de Investigación Sociosanitaria Sobre Cuidadores de Personas Mayores Dependientes; Sociedad Española de Geriatría y Gerontología; IMC—International Marketing & Communication S.A.: Madrid, Spain. http://envejecimiento.csic.es/documentos/documentos/Estudio-Cuidadores-segg.pdf.

[12] Hermida E., Vázquez F.L., Blanco V., Otero P., Torres Á. El Malestar Emocional en los Cuidadores No Profesionales. Proceedings of the 6th International and 11th National Congress of Clinical Psychology.

[13] Pérez Peñaranda A., García Ortiz L., Rodríguez Sánchez E., Losada Baltar A., Porras Santos N., Gómez Marcos M.Á. (2009). Función familiar y salud mental del cuidador de familiares con dependencia. Aten. Primaria.

[14] Baltar A.L. (2006). Estudio e Intervención sobre el Malestar Psicológico de los Cuidadores de Personas con Demencia.

[15] Awan M., Qureshi S., Tariq H., Siddiqi F. (2019). Effects of Cardiopulmonary Conditioning on Body Mass Index, Physical Activity and General Psychological Health of Young Adults. Pak. Heart J..

[16] Ohrnberger J., Fichera E., Sutton M. (2017). The Relationship between Physical and Mental Health: A Mediation Analysis. Soc. Sci. Med..

[17] Biddle S.J.H., Asare M. (2011). Physical Activity and Mental Health in Children and Adolescents: A Review of Reviews. Br. J. Sport. Med..

[18] Dinas P.C., Koutedakis Y., Flouris A.D. (2011). Effects of Exercise and Physical Activity on Depression. Ir. J. Med. Sci..

[19] Dishman R.K., O’Connor P.J. (2009). Lessons in Exercise Neurobiology: The Case of Endorphins. Ment. Health Phys. Act..

[20] Grisel J.E., Bartels J.L., Allen S.A., Turgeon V.L. (2008). Influence of β-Endorphin on Anxious Behavior in Mice: Interaction with EtOH. Psychopharmacology.

[21] Sloan R.A., Sawada S.S., Martin C.K., Church T., Blair S.N. (2009). Associations between Cardiorespiratory Fitness and Health-Related Quality of Life. Health Qual. Life Outcomes.

[22] Denche-Zamorano Á., Muñoz-Bermejo L., Carlos-Vivas J., Mendoza-Muñoz M., Franco-García J.M., Rojo-Ramos J., Vega-Muñoz A., Contreras-Barraza N., Barrios-Fernandez S. (2022). A Cross-Sectional Study about the Associations between Physical Activity Level, Self-Perceived Health Perception and Mental Health in Informal Caregivers of Elderly or People with Chronic Conditions in Spain. Int. J. Environ. Res. Public. Health.

[23] Muñoz-Bermejo L., Villafaina S., Collado-Mateo D., Postigo-Mota S., Adsuar J.C. (2019). Physical Strength Perception of Older Caregivers in Rural Areas. Medicina.

[24] Egan K.J., Hodgson W., Dunlop M.D., Imperatore G., Kirk A., Maguire R. (2021). A Novel Mobile App (“CareFit”) to Support Informal Caregivers to Undertake Regular Physical Activity from Home During and Beyond COVID-19 Restrictions: Co-Design and Prototype Development Study. JMIR Form. Res..

[25] Instituto Nacional de Estadística Metodología de La Encuesta Europea de Salud En España 2014 2014. Ministerio de Sanidad, Consumo y Bienestar Social Encuesta Nacional de Salud de España 2017. https://www.sanidad.gob.es/estadEstudios/estadisticas/encuestaNacional/encuesta2017.htm.

[26] Instituto Nacional de Estadística Metodología de La Encuesta Europea de Salud En España 2020 2020. Sanidad Consumo, M.; Social, B. Encuesta Nacional de Salud 2017 ENSE 2017: Metodología. https://www.ine.es/metodologia/t15/t153041917.pdf.

[27] Shfiezadeh A., Mirzaee A., Heravi-Karimooi M., Rejeh N., Sharif Nia H., Montazeri A. (2019). Anxiety and Depression in Caregivers of Elderly with Alzheimer. Payesh Health Monit..

[28] Ketcher D., Trettevik R., Vadaparampil S.T., Heyman R.E., Ellington L., Reblin M. (2020). Caring for a Spouse with Advanced Cancer: Similarities and Differences for Male and Female Caregivers. J. Behav. Med..

[29] Oechsle K., Ullrich A., Marx G., Benze G., Wowretzko F., Zhang Y., Dickel L.-M., Heine J., Wendt K.N., Nauck F. (2020). Prevalence and Predictors of Distress, Anxiety, Depression, and Quality of Life in Bereaved Family Caregivers of Patients With Advanced Cancer. Am. J. Hosp. Palliat. Care.

[30] Unsar S., Erol O., Ozdemir O. (2021). Caregiving Burden, Depression, and Anxiety in Family Caregivers of Patients with Cancer. Eur. J. Oncol. Nurs. Off. J. Eur. Oncol. Nurs. Soc..

[31] Thomann P., Rousseau A., Valette S., Martin-Hunyadi C., Vogel T., Michel B. (2022). Psychotropic Medication Use by Informal Caregivers of Elderly Patients with Dementia: A French Observational Study. NPG Neurol. Psychiatr. Gériatrie.

[32] Wolf S., Seiffer B., Zeibig J.-M., Welkerling J., Brokmeier L., Atrott B., Ehring T., Schuch F.B. (2021). Is Physical Activity Associated with Less Depression and Anxiety During the COVID-19 Pandemic? A Rapid Systematic Review. Sport. Med..

[33] McDowell C.P., Dishman R.K., Gordon B.R., Herring M.P. (2019). Physical Activity and Anxiety: A Systematic Review and Meta-Analysis of Prospective Cohort Studies. Am. J. Prev. Med..

[34] Baik D., Song J., Tark A., Coats H., Shive N., Jankowski C. (2021). Effects of Physical Activity Programs on Health Outcomes of Family Caregivers of Older Adults with Chronic Diseases: A Systematic Review. Geriatr. Nurs..

[35] Montero-Cuadrado F., Galán-Martín M.Á., Sánchez-Sánchez J., Lluch E., Mayo-Iscar A., Cuesta-Vargas Á. (2020). Effectiveness of a Physical Therapeutic Exercise Programme for Caregivers of Dependent Patients: A Pragmatic Randomised Controlled Trial from Spanish Primary Care. Int. J. Environ. Res. Public. Health.

[36] Doyle K.L., Toepfer M., Bradfield A.F., Noffke A., Ausderau K.K., Andreae S., Pickett K.A. (2021). Systematic Review of Exercise for Caregiver-Care Recipient Dyads: What Is Best for Spousal Caregivers-Exercising Together or Not at All?. Gerontologist.

[37] Valero-Cantero I., Casals C., Corral-Pérez J., Barón-López F.J., Wärnberg J., Vázquez-Sánchez M.Á. (2023). Accelerometer-Measured Physical Activity, Inactivity, and Related Factors in Family Caregivers of Patients with Terminal Cancer. Int. J. Environ. Res. Public. Health.

[38] McAuley E., Elavsky S., Jerome G.J., Konopack J.F., Marquez D.X. (2005). Physical Activity-Related Well-Being in Older Adults: Social Cognitive Influences. Psychol. Aging.

[39] Elavsky S., McAuley E., Motl R.W., Konopack J.F., Marquez D.X., Hu L., Jerome G.J., Diener E. (2005). Physical Activity Enhances Long-Term Quality of Life in Older Adults: Efficacy, Esteem, and Affective Influences. Ann. Behav. Med. Publ. Soc. Behav. Med..

[40] Marshall E., LaCaille R.A., LaCaille L.J., Lee J.E., Peterson E. (2022). Effects of Physical Activity Interventions for Caregivers of Adults: A Meta-Analysis. Health Psychol..

