# Supplementary material for: Depression, Anxiety and Antidepressants and Anxiolytics Use in Spanish Informal Caregivers according to the Physical Activity Frequency: EHSS 2014–2020
Source: Healthcare (Basel). 2023 Mar 30;11(7):990. doi: 10.3390/healthcare11070990 (PMC10094340; doi:10.3390/healthcare11070990)
Supplement: Supplementary file 1 [file healthcare-11-00990-s001.zip › healthcare-2242095-supplementary.pdf]

Table S1. Sample characterisation.

	EHSS 2014					
	Men		Women		Total	
	Median	IQR	Median	IQR	Median	IQR
Age (Years)	52	18	53	17	52	17
PAF	n	%	n	%	n	%
Inactive	296	30.6	608	38.3	904	35.4
Occasional	433	44.8	671	42.3	1104	43.3
Active	102	10.6	149	9.4	251	9.8
Very Active	135	14.0	158	10.0	293	11.5
Hours care/week						
<10 hours	386	40.1	487	30.9	873	34.4
(10 hours, 20 hours]	206	21.4	292	18.5	498	19.6
>20 hours	371	38.5	797	50.6	1168	46.0
Who care?						
Familiar	928	96.1	1472	92.8	2400	94.0
No familiar	38	3.9	114	7.2	152	6.0
	EHSS 2020					
	Men		Women		Total	
	Median	IQR	Median	IQR	Median	IQR
Age (Years)	55	17	55	16	55	17
PAF	n	%	n	%	n	%
Inactive	265	30.7	477	34.0	742	32.7
Occasional	331	38.4	574	40.9	905	39.9
Active	104	12.1	146	10.4	250	11.0
Very Active	163	18.9	208	14.8	371	16.4
Hours care/week						
<10 hours	327	38.2	453	32.5	780	34.7
(10 hours, 20 hours]	159	18.6	279	20.0	438	19.5
>20 hours	370	43.2	662	47.5	1032	45.9
Who care?						
Familiar	844	97.9	1336	95.1	2180	96.1
No familiar	19	2.2	69	4.9	88	3.9

Data presented in absolute and relative values; IQR (Interquartile Range); PAF (Physical Activity Frequency); EHSS (European Health Survey in Spain)

Table S2. Depression, anxiety, antidepressants and anxiolitics use prevalence in Spanish no-formal caregivers according to the physical activiy frequency. EHSS2014-2020

	Inactive		Occasional		Active		Very active					
**2014**												
	n=904	(%)	n=1104	(%)	n=251	(%)	n=293	(%)	x^2	df	p	CC
Depression	183	(20.2)a	157	(14.2)b	16	(6.4)c	27	(9.2)bc	42.3	3	<0.001	0.128
No depression	721	(79.8)a	947	(85.8)b	235	(93.6)c	266	(90.8)bc				
Anxiety	168	(18.6)a	149	(13.5)b	12	(4.8)c	23	(7.8)bc	43.4	3	<0.001	0.129
No anxiety	736	(81.4)a	955	(86.5)b	239	(95.2)c	270	(92.2)bc				
Antidepressant	98	(10.8)a	80	(7.2)b	10	(4.0)bc	7	(2.4)c	29.6	3	<0.001	0.107
No Antidepressants	806	(89.2)a	1024	(92.8)b	241	(96.0)bc	286	(97.6)c				
Anxiolitics	193	(21.3)a	174	(15.8)b	30	(12.0)bc	22	(7.5)c	37.0	3	<0.001	0.119
No anxiolitics	711	(78.7)a	930	(84.2)b	221	(88.0)bc	271	(92.5)c				
**2020**												
	Inactive		Occasional		Active		Very active					
	n=742	(%)	n=905	(%)	n=250	(%)	n=371	(%)	x^2	df	p	CC
Depression	120	(16.2)a	98	(10.8)b	21	(8.4)b	29	(7.8)b	22.9	3	<0.001	0.100
No depression	622	(83.8)a	807	(89.2)b	229	(91.6)b	342	(92.2)b				
Anxiety	109	(14.7)a	102	(11.3)ab	22	(8.8)ab	25	(6.7)b	17.7	3	<0.001	0.088
No anxiety	633	(85.3)a	803	(88.7)ab	228	(91.2)ab	346	(93.3)b				
Antidepressant	72	(9.7)a	59	(6.5)ab	10	(4.0)bc	10	(2.7)b	23.3	3	<0.001	0.101
No Antidepressants	670	(90.3)a	846	(93.5)ab	240	(96.0)bc	361	(97.3)b				
Anxiolitics	136	(18.3)a	126	(13.9)a	18	(7.2)b	26	(7.0)b	36.9	3	<0.001	0.126
No anxiolitics	606	(81.7)a	779	(86.1)a	232	(92.8)b	345	(93.0)b				

n (Participants); % (Percentage); x^2 (Pearson Chi-Square); df (degree freedom); p (p-value from Chi-Square test); CC (Contingency coefficient); * (Different letters means significant differences of proportions in pairwise z-test for independent proportions, p<0.05).

Table S3. Logarithmic binary regression model for depression, anxiety, antidepressants and anxiolitics use. EHSS2014

EHSS 2014

Depression

	B	S.E.	Wald	df	Sig.	Exp(B)	95% C.I.for EXP(B)	
							Lower	Upper
Sex (Men)	0.734	0.132	31.120	1	0.000	2.084	1.610	2.697
Age	0.014	0.004	11.176	1	0.001	1.014	1.006	1.023
PAF: Inactive			24.167	3	0.000			
Occasional	-0.357	0.122	8.565	1	0.003	0.699	0.551	0.889
Active	-1.099	0.275	15.921	1	0.000	0.333	0.194	0.572
Very active	-0.655	0.224	8.579	1	0.003	0.519	0.335	0.805
Hours/week: <10h			7.435	2	0.024			
(10h, 20h]	0.259	0.173	2.253	1	0.133	1.296	0.924	1.817
<20h	0.385	0.141	7.432	1	0.006	1.469	1.114	1.937
Who? Familiar	0.461	0.219	4.452	1	0.035	1.586	1.033	2.435
Constant	-2.988	0.281	113.418	1	0.000	0.050		

Anxiety

	B	S.E.	Wald	df	Sig.	Exp(B)	95% C.I.for EXP(B)	
							Lower	Upper
Sex (Men)	0.843	0.139	36.686	1	0.000	2.324	1.769	3.053
Age	0.009	0.004	4.157	1	0.041	1.009	1.000	1.018
PAF: Inactive			27.330	3	0.000			
Occasional	-0.291	0.125	5.410	1	0.020	0.748	0.585	0.955
Active	-1.426	0.324	19.430	1	0.000	0.240	0.127	0.453
Very active	-0.753	0.239	9.970	1	0.002	0.471	0.295	0.751
Hours/week: <10h			7.771	2	0.021			
(10h, 20h]	0.220	0.179	1.509	1	0.219	1.246	0.877	1.770
<20h	0.401	0.145	7.689	1	0.006	1.494	1.125	1.984
Constant	-2.863	0.288	99.172	1	0.000	0.057		

Antidepressants

	B	S.E.	Wald	df	Sig.	Exp(B)	95% C.I.for EXP(B)	
							Lower	Upper
Sex (Men)	0.821	0.186	19.468	1	0.000	2.273	1.578	3.274
PAF: Inactive			18.717	3	0.000			
Occasional	-0.356	0.161	4.920	1	0.027	0.700	0.511	0.959
Active	-0.899	0.343	6.850	1	0.009	0.407	0.208	0.798
Very active	-1.390	0.400	12.069	1	0.001	0.249	0.114	0.546
Hours/week: <10h			18.506	2	0.000			
(10h, 20h]	0.196	0.254	0.597	1	0.440	1.217	0.740	2.002
<20h	0.755	0.192	15.462	1	0.000	2.128	1.460	3.100
Constant	-3.206	0.235	186.767	1	0.000	0.041		

Anxiolitics

	B	S.E.	Wald	df	Sig.	Exp(B)	95% C.I.for EXP(B)	
							Lower	Upper
Sex (Men)	0.800	0.128	39.060	1	0.000	2.225	1.732	2.860
Age	0.025	0.004	33.799	1	0.000	1.025	1.016	1.033
PAF: Inactive			17.092	3	0.001			
Occasional	-0.288	0.119	5.843	1	0.016	0.750	0.593	0.947
Active	-0.441	0.220	4.017	1	0.045	0.644	0.418	0.990
Very active	-0.885	0.241	13.441	1	0.000	0.413	0.257	0.662
Hours/week: <10h			8.364	2	0.015			
(10h, 20h]	0.335	0.166	4.064	1	0.044	1.398	1.009	1.936

<20h	0.387	0.137	8.030	1	0.005	1.473	1.127	1.926
Constant	-3.543	0.281	159.075	1	0.000	0.029		

B: Understandarized beta; SE: Standard error of the regression; Wald: Wald Chi-Squared Test; df: Degrees of freedom; Sig: Statistical significance; Exp: Exponential regression; CI: Confidence Interval

Table S4. Logarithmic binary regression model for depression, anxiety, antidepressants and anxiolitics use (EHSS2020).

EHSS 2020								
Depression								
	B	S.E.	Wald	df	Sig.	Exp(B)	95% C.I.for EXP(B)	
							Lower	Upper
Sex (Men)	0.501	0.147	11.547	1	0.001	1.650	1.236	2.203
Age	0.014	0.005	7.128	1	0.008	1.014	1.004	1.024
PAF: Inactive			15.768	3	0.001			
Occasional	-0.467	0.149	9.785	1	0.002	0.627	0.468	0.840
Active	-0.626	0.252	6.155	1	0.013	0.535	0.326	0.877
Very active	-0.633	0.222	8.125	1	0.004	0.531	0.344	0.821
Hours/week: <10h			8.707	2	0.013			
(10h, 20h]	0.329	0.201	2.677	1	0.102	1.390	0.937	2.062
<20h	0.487	0.165	8.702	1	0.003	1.627	1.178	2.249
Who? Familiar	0.834	0.277	9.043	1	0.003	2.302	1.337	3.965
Constant	-3.134	0.334	88.092	1	0.000	0.044		
Anxiety								
	B	S.E.	Wald	df	Sig.	Exp(B)	95% C.I.for EXP(B)	
							Lower	Upper
Sex (Men)	0.778	0.156	24.950	1	0.000	2.177	1.604	2.954
PAF: Inactive			14.382	3	0.002			
Occasional	-0.284	0.149	3.624	1	0.057	0.753	0.562	1.008
Active	-0.533	0.248	4.621	1	0.032	0.587	0.361	0.954
Very active	-0.802	0.233	11.833	1	0.001	0.449	0.284	0.708
Constant	-2.313	0.158	213.230	1	0.000	0.099		
Antidepressants								
	B	S.E.	Wald	df	Sig.	Exp(B)	95% C.I.for EXP(B)	
							Lower	Upper
Sex (Men)	0.831	0.205	16.393	1	0.000	2.296	1.536	3.434
Age	0.015	0.006	5.252	1	0.022	1.015	1.002	1.028
PAF: Inactive			18.076	3	0.000			
Occasional	-0.445	0.185	5.780	1	0.016	0.641	0.446	0.921
Active	-0.853	0.348	6.006	1	0.014	0.426	0.215	0.843
Very active	-1.236	0.346	12.753	1	0.000	0.291	0.148	0.573
Constant	-3.665	0.429	72.932	1	0.000	0.026		
Anxiolitics								
	B	S.E.	Wald	df	Sig.	Exp(B)	95% C.I.for EXP(B)	
							Lower	Upper
Sex (Men)	0.573	0.140	16.817	1	0.000	1.773	1.348	2.331
Age	0.025	0.005	26.792	1	0.000	1.025	1.016	1.035
PAF: Inactive			25.709	3	0.000			
Occasional	-0.324	0.137	5.580	1	0.018	0.723	0.553	0.946
Active	-0.933	0.265	12.393	1	0.000	0.393	0.234	0.661
Very active	-0.937	0.227	17.013	1	0.000	0.392	0.251	0.611
Constant	-3.328	0.319	108.544	1	0.000	0.036		

B: Understandarized beta; SE: Standard error of the regression; Wald: Wald Chi-Squared Test; df: Degrees of freedom; Sig: Statistical significance; Exp: Exponential regression; CI: Confidence Interval; Hours/Week (Hours per week dedicated to the care of dependent person(s)); Who? (The person(s) you are caring for are: family or non-family).

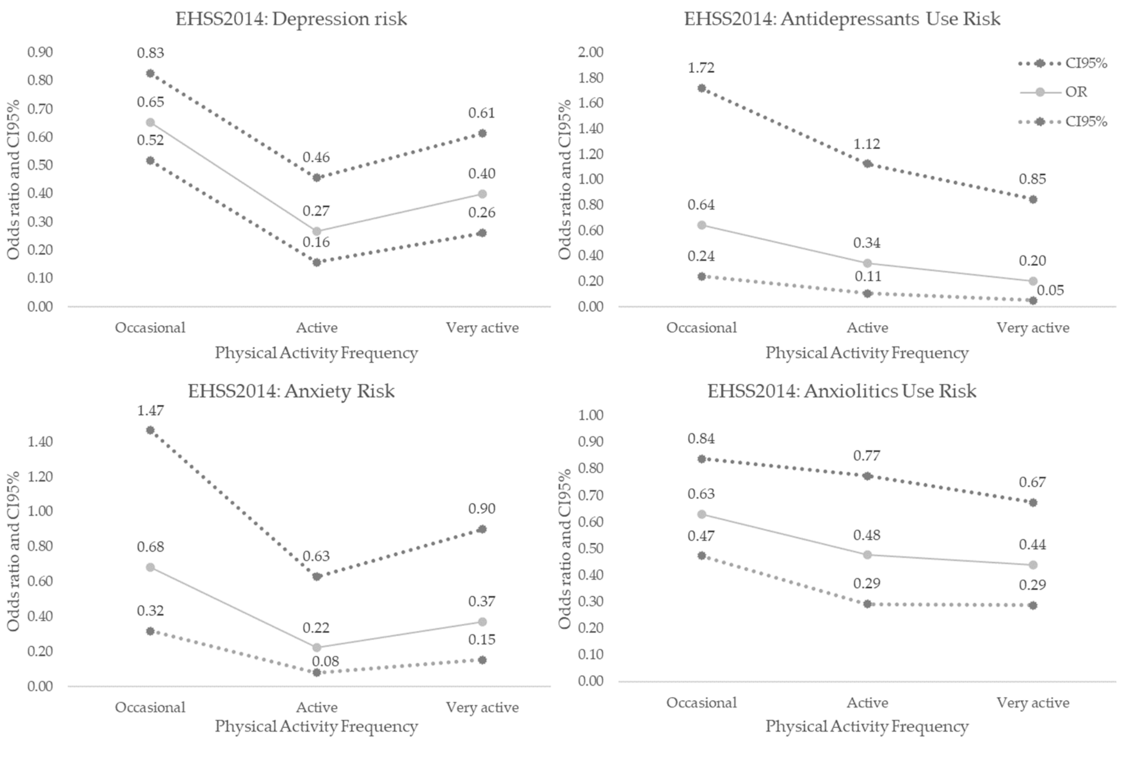

Figure S1. Risk probability of depression, anxiety, antidepressant use and anxiolytic use in the inactive population according to physical activity frequency in Spanish non-formal caregivers from EHSS 2014 (OR: Odd ratios; CI: Confidence Interval.

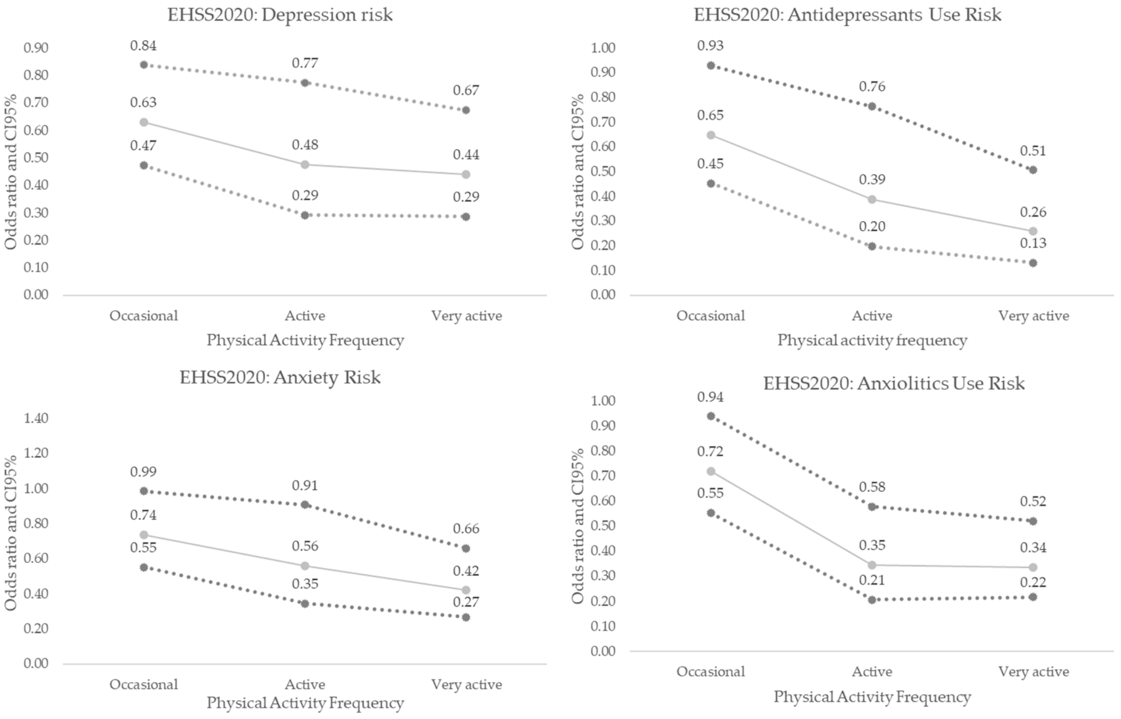

Figure S2. Odds ratios of depression, anxiety, antidepressant and anxiolytic use in the inactive population, based on physical activity frequency in Spanish no formal caregivers from EHSS 2014 (OR: Odd ratios; CI: Confidence Interval).
